# Genetic screens reveal new targetable vulnerabilities in BAP1-deficient mesothelioma

**DOI:** 10.1016/j.xcrm.2022.100915

**Published:** 2023-01-18

**Authors:** Gaurav Kumar Pandey, Nick Landman, Hannah K. Neikes, Danielle Hulsman, Cor Lieftink, Roderick Beijersbergen, Krishna Kalyan Kolluri, Sam M. Janes, Michiel Vermeulen, Jitendra Badhai, Maarten van Lohuizen

**Affiliations:** 1 Division of Molecular Genetics, The Netherlands Cancer Institute, Plesmanlaan 121, 1066CX Amsterdam, the Netherlands; 2 Department of Molecular Biology, Faculty of Science, Radboud Institute for Molecular Life Sciences, Radboud University Nijmegen, Nijmegen, the Netherlands; 3 Division of Molecular Carcinogenesis, NKI Robotics and Screening Center, The Netherlands Cancer Institute, Amsterdam, the Netherlands; 4 Oncode Institute, Utrecht, the Netherlands; 5 Lung for Living Research Centre, UCL Respiratory, University College London, Rayne Building, London, UK

**Keywords:** BAP1, mesothelioma, uveal melanoma, Polycomb, EZH2, mevalonate, combination therapy, targeted therapy, preclinical models

## Abstract

More than half of patients with malignant mesothelioma show alterations in the *BAP1* tumor-suppressor gene. Being a member of the Polycomb repressive deubiquitinating (PR-DUB) complex, *BAP1* loss results in an altered epigenome, which may create new vulnerabilities that remain largely unknown. Here, we performed a CRISPR-Cas9 kinome screen in mesothelioma cells that identified two kinases in the mevalonate/cholesterol biosynthesis pathway. Furthermore, our analysis of chromatin, expression, and genetic perturbation data in mesothelioma cells suggests a dependency on PR complex 2 (PRC2)-mediated silencing. Pharmacological inhibition of PRC2 elevates the expression of cholesterol biosynthesis genes only in *BAP1*-deficient mesothelioma, thereby sensitizing these cells to the combined targeting of PRC2 and the mevalonate pathway. Finally, by subjecting autochthonous *Bap1*-deficient mesothelioma mice or xenografts to mevalonate pathway inhibition (zoledronic acid) and PRC2 inhibition (tazemetostat), we demonstrate a potent anti-tumor effect, suggesting a targeted combination therapy for *Bap1*-deficient mesothelioma.

## Highlights

• CRISPR-Cas9 kinome screen identifies new dependencies in BAP1-deficient mesothelioma
• Mesothelioma cells lacking BAP1 are vulnerable to mevalonate pathway inhibition
• Mevalonate pathway combined with EZH2 inhibition is potent against BAP1-mutated tumors
• This combination is well tolerated and prolongs survival in *in vivo* mesothelioma models

## Graphical abstract

**Figure:**
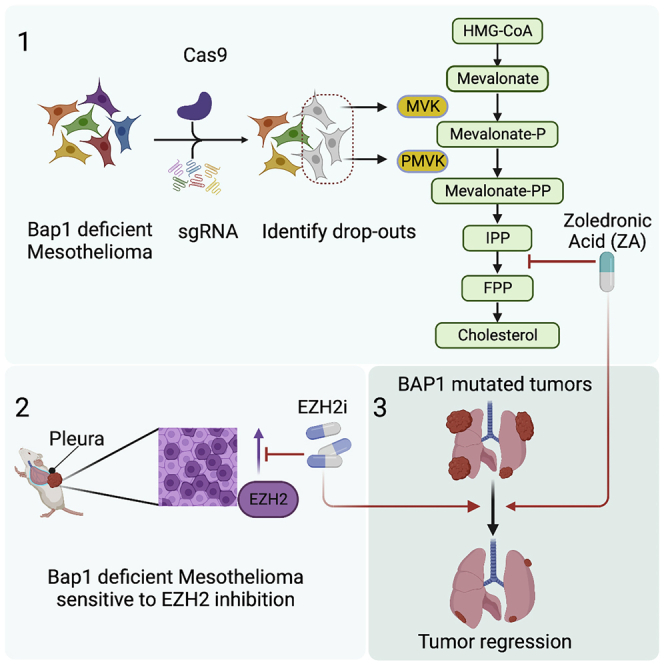

## Teaser

BAP1 mutant mesothelioma tumors have a poor prognosis and few therapeutic options. Here, Pandey and Landman et al. show that the combined targeting of mevalonate pathway and EZH2 inhibition effectively kills tumor cells and prolongs the survival of Bap1-deficient mice with mesothelioma.

## Introduction

Mesothelioma is a highly aggressive cancer of serosal surfaces linked to asbestos exposure and is fatal in nearly all cases. The median survival of affected individuals ranges from only 12 up to 18 months after diagnosis.^1^ Currently approved frontline therapies, including immunotherapy (nivolumab plus ipilimumab) and chemotherapy (cisplatin plus pemetrexed), modestly extend the overall survival by a few months only.^2^ Therefore, there is an urgent unmet need for novel therapeutic strategies based on biomarker stratification of patients and formulating new tailored therapies.

*BAP1*, a known tumor suppressor, is one such candidate biomarker that is mutated or deleted in a significant fraction of malignant mesothelioma (60%–70%) as well as in other cancers such as uveal melanoma (43%) and renal cell carcinoma (23%).^3^^,^^4^^,^^5^^,^^6^^,^^7^^,^^8^^,^^9^^,^^10^ As a catalytic subunit of the Polycomb repressive deubiquitinating (PR-DUB) complex, BAP1 deubiquitinates PR complex 1 (PRC1)-mediated H2AK119ub1 and opposes the gene function of PRC1 and PRC2.^11^^,^^12^^,^^13^^,^^14^ We recently described new mouse models of mesothelioma where *Bap1* deletion accompanied with alterations in genetic drivers *Nf2* and *Cdkn2ab* results in fast and aggressive mesothelioma.^15^ This aggressiveness is associated with elevated signaling of PI3K and MAPK pathways.^16^ In addition, *Bap1*-deficient mesothelioma is dependent on increased Polycomb repression, creating a *Bap1*-loss-specific vulnerability.^15^^,^^17^ Thus, our *Bap1* mouse models, which closely mimic human malignant mesothelioma, provide an ideal genetic setting for identifying synthetic lethal interactions and investigating the *BAP1*-polycomb connections in cancer.

CRISPR-based genetic screens are being extensively used to identify synthetic lethal interactions and aid in the development of new combination therapies for treating cancers.^18^^,^^19^ In a forward genetic screen using a kinome CRISPR library, we identify that *Bap1-*deficient mesothelioma cells are sensitive to the loss of kinases belonging to a major metabolic pathway involved in mevalonate and cholesterol biosynthesis. Exploiting this vulnerability pharmacologically, we show that mesothelioma cells lacking BAP1 are more susceptible to the mevalonate pathway inhibitor zoledronic acid (ZA), identifying another *Bap1*-loss-specific vulnerability. Next, we sought to identify key target genes of BAP1 commonly regulated by PRC2 repression in both mouse and human mesothelioma. Finally, by combined targeting of both vulnerabilities, we demonstrate a potent anti-tumor effect, suggesting a new targeted combination therapy for *BAP1*-deficient mesothelioma.

## Results

### Focused CRISPR-Cas9 kinome screen identifies mevalonate kinase (Mvk) and phosphomevalonate kinase (Pmvk) dependencies in *Bap1*-deficient mesothelioma cells

**Figure 1:**
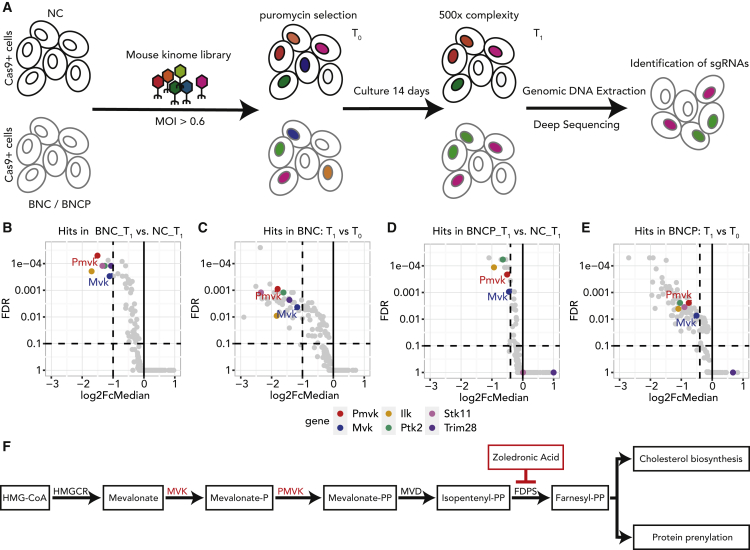
Focused CRISPR-Cas9 kinome screen identifies Mvk and Pmvk dependencies in *Bap1*-deficient mesothelioma cells (A) Schematic representation of experimental workflow of the dropout kinome CRISPR-Cas9 screen in *Bap1*-deficient (BNC/BNCP) and proficient (NC) mesothelioma cell lines. (B–E) Volcano plots showing the significantly dropped-out genes, (B) comparing BNC and NC at T_1_ (FDR ≤ 0.1, log2 fold change [FC] ≤ −1); (C) comparing T_1_ with T_0_ for BNC cells (FDR ≤ 0.1, log2FC ≤ −1); (D) comparing BNCP and NC at T_1_ (FDR ≤ 0.1, log2FC ≤ −0.4); and (E) comparing T_1_ with T_0_ for BNCP cells (FDR ≤ 0.1, log2FC ≤ −0.4). The top 6 hits from the screen are highlighted in color. (F) Simplified schematic representation of the mevalonate pathway where primary hits from our screen are highlighted in red color. The inhibitor of FDPS, zoledronic acid, is highlighted as well. See also Figure S1.

Oncogenic signaling pathways such as PI3K, MAPK, and receptor tyrosine kinases are frequently activated and altered in malignant mesothelioma.^4^^,^^20^ With the lack of efficient targeted therapy for mesothelioma and kinase inhibition being one of the most pharmacologically tractable therapeutic strategies, we performed a dropout screen against kinases in *Bap1*-proficient and -deficient settings. Toward this, we have utilized three mesothelioma mouse model derived cell lines, referred to as NC (*Nf2*^−/−^, *Cdkn2ab*^−/−^), BNC (*Bap1*^−/−^, *Nf2*^−/−^, *Cdkn2ab*^−/−^), and BNCP (*Bap1*^−/−^, *Nf2*^−/−^, *Cdkn2ab*^−/−^, *Tp53*^−/−^), which allow us to discover *Bap1-*loss-associated dependencies in a defined genetic background.^15^ We utilized a mouse kinome knockout library (Brie) containing single guide RNAs (sgRNAs) targeting 713 mouse kinases with four guides per target.^21^ The transduced mesothelioma cells were selected by puromycin for 3 days (T_0_) and maintained for 14 days (T_1_) at 500× representation (Figure 1A). The sgRNAs from T_0_ and T_1_ were amplified by a two-step PCR and subsequently quantified by next-generation sequencing. The detailed methodology for the screen and data analysis is described in the STAR Methods.

To identify dropouts specific to *Bap1*-deficient cell lines compared with *Bap1*-proficient ones, a DESeq2 analysis on the sgRNA level followed by MAGeCK’s robust rank analysis (RRA) was performed with two replicates for each condition (Figure S1A). The hits were selected based on three thresholds at sgRNA level: log10 base mean ≥100, false discovery rate (FDR) ≤0.1, and log2 fold change ≤−1. Applying these criteria, we identified six kinases significantly depleted in *Bap1*-deficient cells compared with *Bap1*-proficient cells (Figures 1B, 1C, and S1B). Interestingly, our top hits include two kinases belonging to the same cellular pathway, i.e., *Mvk* and *Pmvk*. Both these genes are also negatively selected in an independent BNCP line in which, besides *Bap1*, *Trp53* is deleted, reiterating our primary observations (Figures 1D and 1E). MVK and PMVK are members of the mevalonate pathway, an important metabolic pathway in which mevalonate is converted into precursor molecules for essential metabolites involved in processes such as the synthesis of cholesterol, dolichol, and ubiquinone (Figure 1F).^22^ Interestingly, a proteomics study by Baughman et al. has earlier demonstrated the role of Bap1 as a metabolic regulator in the liver and pancreas of the mouse.^23^ Specifically, acute deletion of *Bap1* in mouse liver profoundly elevated cholesterol biosynthesis metabolites, including Mvk. Taken together, these findings suggest that mesothelioma cells with a loss-of-function mutation in *Bap1* are dependent on metabolic pathways such as the mevalonate pathway, which may eventually contribute to the pathogenesis of *Bap1*-deficient mesothelioma.

### Loss of BAP1 renders mesothelioma cells sensitive to mevalonate pathway inhibition

**Figure 2:**
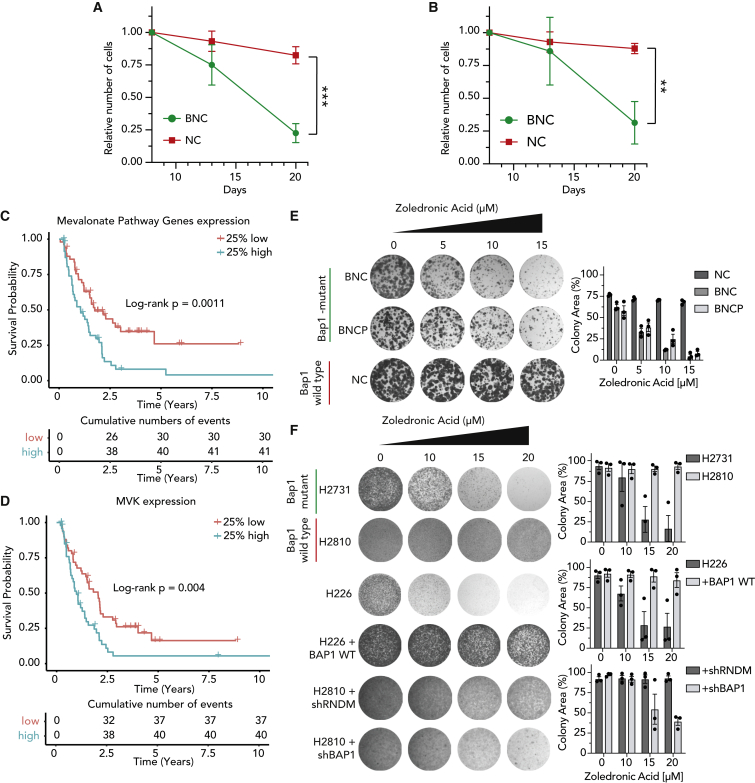
Loss of *BAP1* renders mesothelioma cells sensitive to mevalonate pathway inhibition (A and B) Competitive growth assay showing a decrease in cell fitness upon expression of GFP-tagged gRNA targeting *Pmvk* (A) and *Mvk* (B) in BNC versus NC cells over 3 weeks. Data were normalized against day 8 after transfection (mean ± SD; n = 3 independent experiments). p values were determined by two-tailed unpaired Student’s t test; ∗p < 0.05, ∗∗p < 0.01, and ∗∗∗p < 0.001. (C) Kaplan-Meier curve indicating overall survival (OS) of patients with mesothelioma based on expression of mevalonate pathway genes. Top quartile (25% of patients with highest expression) of mevalonate pathway gene expression versus bottom quartile (25% of patients with lowest expression). The graph depicts p value obtained using the log rank test. (D) Kaplan-Meier curve indicating OS of patients (same cohort as in C) based on expression of MVK; log rank test. (E and F) Colony-formation assays and quantifications showing sensitivity of BAP1-deficient mouse (E) and human (F) mesothelioma cells to zoledronic acid (μM) treatment compared with BAP1-proficient cell lines; representative data shown from three independent experiments. Quantification data are mean ± SEM, n = 3 independent experiments. See also Figure S2.

We used competitive growth assays to validate the dependency of *Bap1*-proficient and -deficient mouse cells on *Pmvk* or *Mvk*. For this, sgRNAs against *Pmvk* or *Mvk* were cloned in a GFP-tagged vector, and the number of GFP-expressing cells was analyzed over time (Figure S2A). We observe that cells with GFP-tagged sgRNAs were significantly more depleted in *Bap1*-null mouse cells (BNC) than *Bap1* wild-type (WT) cell lines (NC) (Figures 2A, 2B, and S2B–S2G). As Baughmann et al. have shown that loss of *Bap1* is directly linked to elevated expression of mevalonate pathway genes in mouse liver, we checked whether this pathway is associated with disease outcome in mesothelioma.^23^ A cohort of 197 patients with mesothelioma was subdivided into quartiles based on expression of mevalonate pathway genes and analyzed for survival probability (Figure S2H). We observed that patients with the 25% highest expression of mevalonate pathway genes showed significantly poorer survival compared with patients with the 25% lowest expression (p = 0.0011; Figure 2C).^4^ Furthermore, the elevated expression of *MVK* is also associated with poor survival among patients with mesothelioma (p = 0.004; Figure 2D). These observations support our findings from the CRISPR screen and suggest that targeting kinases of the mevalonate pathway could serve as a therapeutic option for mesothelioma.

Surprisingly, there are no specific inhibitors available for MVK or PMVK. Therefore, we sought other clinically relevant ways to inhibit the mevalonate pathway. ZA, a third-generation bisphosphonate, is an inhibitor of farnesyl pyrophosphate synthase. This enzyme acts downstream of MVK*,* and ZA is given to patients with osteoporosis and bone metastasis in the clinic.^24^^,^^25^^,^^26^^,^^27^ Keeping in mind the extensive use of ZA in the clinic with low toxicity and a possible drug-repurposing application, we aimed to test its efficacy on mouse mesothelioma cell lines. The long-term colony-formation assays and IC50 curves with ZA treatment in mouse mesothelioma cell lines show that *Bap1*-deficient cell lines are more sensitive to ZA treatment than the *Bap1* WT cell line (Figures 2E and S2I). We extended this observation to human mesothelioma cell lines and observed that *BAP1* mutant human cell lines are more sensitive to ZA treatment than *BAP1* WT human cell lines (Figure 2F). Moreover, the *BAP1-*status-specific sensitivity to ZA is affirmed by the loss of sensitivity in the *BAP1*-negative H226 cell line upon reexpression of BAP1 protein (H226 plus *BAP1* WT) and the acquired sensitivity upon loss of *BAP1* by short hairpin RNA (shRNA) *BAP1* (Figure 2F). Collectively, our data show that *BAP1-*deficient mesothelioma cells are vulnerable to mevalonate pathway inhibition, resulting in higher sensitivity to ZA treatment.

### BAP1 loss induces PRC2-mediated transcriptional repression in mouse and human mesothelioma

BAP1 functions as a deubiquitinase removing the H2AK119ub1 PRC1-mediated chromatin mark, and its regulatory relationship with PRCs has been investigated in various cell systems.^15^^,^^17^^,^^28^^,^^29^^,^^30^ In order to investigate the impact of BAP1 loss on the epigenome, its consequence on the mesothelioma transcriptome, and the connection with the observed sensitivity to mevalonate pathway, we have studied the BAP1-loss-associated chromatin and expression changes in mouse and human mesothelioma.

**Figure 3:**
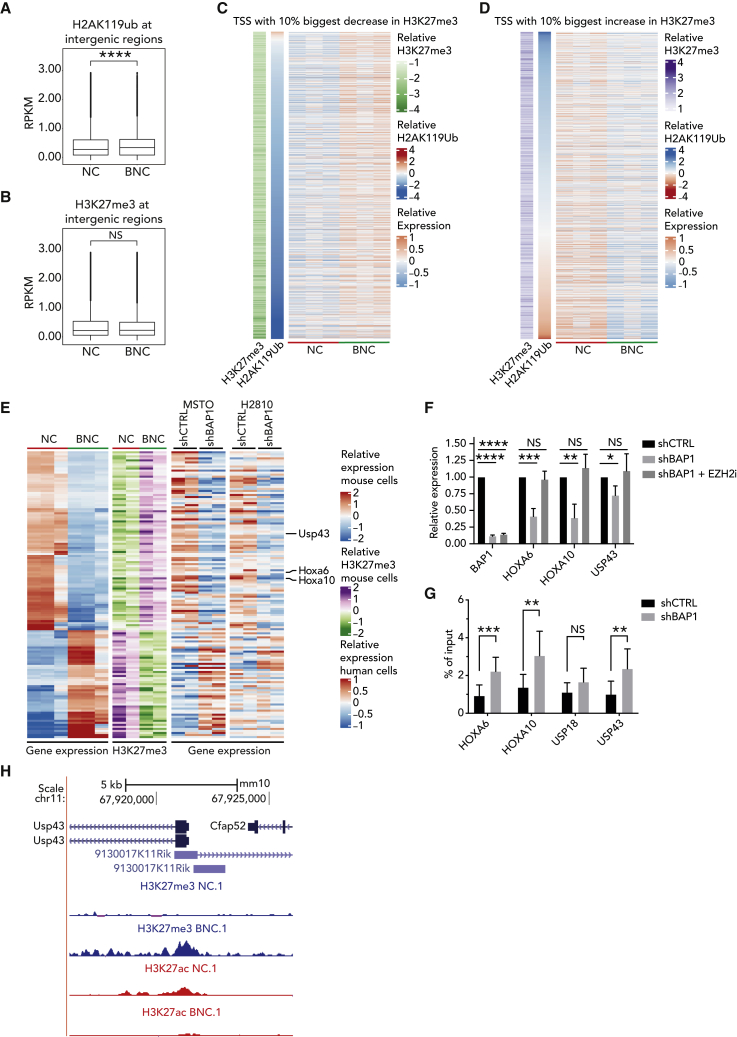
BAP1 loss induces PRC2-mediated transcriptional repression in mouse and human mesothelioma (A) Boxplots representing ChIP-seq RPKM levels in the NC and BNC cells at intergenic regions for H2AK119ub1, showing a significant increase in the relative levels of H2AK119ub1 in BNC cells. (B) Boxplots representing ChIP-seq RPKM levels in the NC and BNC cells at intergenic regions for H3K27me3. (C) Heatmap representing gene expression, as well as changes in H3K27me3 and H2AK119ub ChIP signal at the top 10 percent of genes with the biggest decrease in H3K27me3 occupation at TSS ± 5 kb between NC and BNC cells (n = 3, independent samples per group). (D) Similar to (E) but for the top 10 percent of genes with the biggest increase in H3K27me3 occupation. (E) Heatmap of genes (n = 143) that are differentially expressed in NC and BNC cells (n = 3, biologically independent samples per group) with corresponding chromatin profile of H3K27me3 histone marks. The corresponding expression changes upon shRNA-mediated knockdown of *BAP1* (sh*BAP1*) versus random shRNA control in two human mesothelioma cells is also presented (n = 2 biologically independent samples per group). (F) qPCR measurement of *HOXA6*, *HOXA10*, and *USP43* upon doxycycline-induced shRNA knockdown of *BAP1* (72 h) relative to random shRNA control in H2810 mesothelioma cells (mean ± SD; n = 3 independent experiments). (G) ChIP-qPCR showing H3K27me3 enrichment upon doxycycline-induced downregulation of *BAP1* (72 h). The relative enrichment of H3K27me3 over input at *HOXA6*, *HOXA10*, and *USP43* promoters is presented (mean ± SD; n = 3 independent experiments). (H) Genome browser snapshot of ChIP-seq track at the promoter region of *Usp43* in the NC and BNC mesothelioma cells showing concomitant loss of H3K27me3 and gain of H3K27ac due to loss of Bap1. p values in (A) and (B) were determined by using paired samples Wilcoxon test and in (F) and (G) by using two-tailed unpaired Student’s *t* test; ∗p < 0.05, ∗∗p < 0.01, ∗∗∗p < 0.001, and ∗∗∗∗p < 0.0001. See also Figure S3.

To explore in depth the consequences of *Bap1* loss on H2AK119ub1 and H3K27me3 occupancy, we reanalyzed the chromatin profiles of BNC (*Bap1*-deficient) and NC (*Bap1*-proficient) mouse mesothelioma cells.^15^ The relative level of H2AK119ub1 showed a significant increase in BNC cells (Figure 3A) at the intergenic regions as reported previously in embryonic stem cells (ESCs) and the IST-MES2 human mesothelioma cell line by Conway et al*.*^29^ This increase at intergenic regions could be due to redistribution of chromatin mark H2AK119ub1 upon Bap1 loss. A similar trend, however not significant, is observed for H3K27me3 levels as well (Figure 3B). Interestingly, the H3K27me3 levels do show a relative increase at the promoter regions (Figure S3A).

Next, we studied how the relative changes in H3K27me3 and H2AK119ub1 levels influence gene expression. To this end, we looked at the genes with the 10% biggest increase and decrease in H3K27me3, together with the H2AK119ub1 signal at the same transcription start site (TSS) with relative gene expression (Figures 3C and 3D). A similar analysis was also performed for H2AK119ub1 and plotted against the H3K27me3 signal (Figures S3B and S3C). In addition, we found similar results by performing regression and partial correlation analysis (Figures S3D–S3G). This clearly indicates that the expression pattern follows the dynamics of the H3K27me3 mark rather than H2AK119ub1, suggesting a relatively greater effect of the PRC2-mediated silencing on gene expression in *Bap1*-deficient mesothelioma. To further examine this, we analyzed the set of genes whose expression was consistent with concomitant changes of epigenetic marks (H3K27me3 and H3K27ac) at their promoters. We found 285 genes that were differentially expressed (absolute log2 fold change > 2 and adjusted p [padj] < 0.01) between NC and BNC mesothelioma corresponding to the chromatin profile at their promoter regions (Figure S3H).

To test if our observations hold true in human mesothelioma and whether these changes can be attributed to BAP1 only, we generated an inducible shRNA system (shCTRL or sh*BAP1*) and used this to downregulate *BAP1* in two human mesothelioma cell lines (MSTO and H2810). We identified a set of 143 genes (absolute log2 fold change > 2 and padj < 0.01) that are significantly deregulated in a manner consistent with chromatin changes in mouse mesothelioma and showing matching gene expression in human mesothelioma due to loss of *BAP1* only (Figure 3E). The majority of these genes are downregulated (88 genes), whereas only 53 genes are upregulated. We validated the effect of *BAP1* loss on several genes obtained from our analysis using inducible shRNA and synthetic gRNAs in human mesothelioma cell lines (Figures 3F and S3I). Additionally, using inducible shRNAs, we have performed H3K27me3 chromatin immunoprecipitation (ChIP)-qPCR at promoters of these genes 72 h post-induction with doxycycline and observed a significant increase of the chromatin mark upon induction of *BAP1* loss (Figure 3G). Finally, we used EZH2 inhibition to demonstrate that reduced expression of those genes in the presence of *BAP1* depletion can be rescued by targeting PRC2 (Figure 3F). The expression and chromatin analyses at genes such as *USP43*, *HOXA6*, *HOXA10*, and *USP18* demonstrate that these genes are downregulated via PRC2-mediated silencing in *BAP1*-deficient mesothelioma.

Thus, our analysis and experimental validation across mouse and human mesothelioma cells clearly identify a set of genes regulated via the BAP1-PRC2 axis. We hypothesized that some of these genes, epigenetically repressed due to BAP1 loss, may have tumor-suppressive properties. Indeed, we observed genes such as *USP43*, whose tumor-suppressive roles have been reported earlier, to be silenced due to increased enrichment of H3K27me3 in mouse models lacking *Bap1* (Figure 3H).^31^ Interestingly, lower expression of *USP43* is significantly associated with poor survival outcomes in patients with mesothelioma (Figure S3J).

To connect *Bap1* loss and mevalonate pathway dependency, we examined the relative gene expression of mevalonate pathway genes in mouse and human cell lines. We observe that the majority of these genes are significantly upregulated upon *BAP1* loss in human mesothelioma cells (Figure S3K). A subset of these genes were also significantly upregulated in mouse cell lines (Figure S3L). However, the mevalonate pathway does not appear to be significantly enriched in the H3K27me3 or H2AK119ub1 data, suggesting a potential indirect regulation by these marks.

Overall, our results indicate PRC2-mediated epigenetic silencing could be a driver of the altered transcriptome in *BAP1*-deficient mesothelioma and may influence upregulation of the mevalonate pathway. Furthermore, the observed PRC2 dependency could be the plausible reason behind the sensitivity to EZH2 inhibitors shown in earlier studies.^15^

### BAP1-altered tumor cells are highly sensitive to combined inhibition of EZH2 and the mevalonate pathway

**Figure 4:**
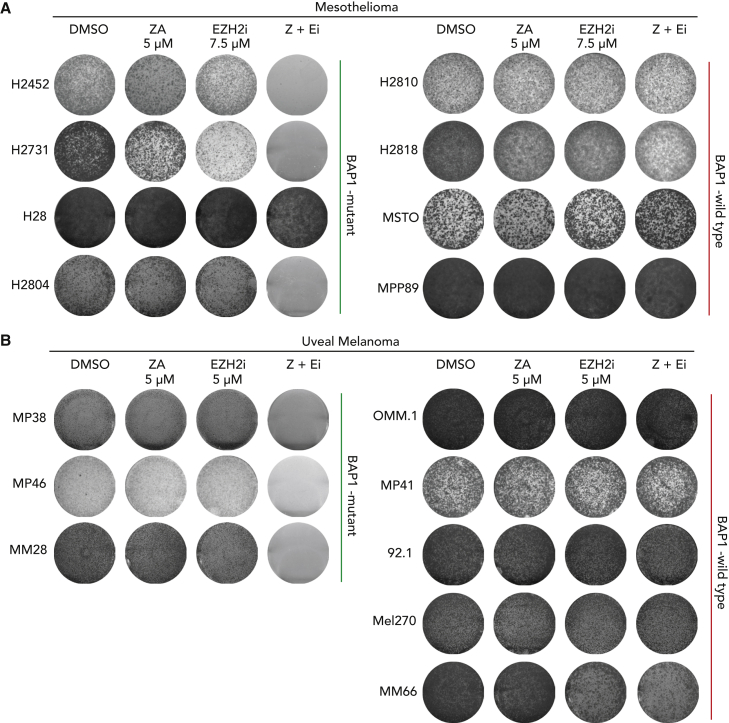
Combination treatment shows a strong selective sensitivity against BAP1-deficient cells *in vitro* (A) Colony-formation assays of human mesothelioma cell lines to zoledronic acid, GSK126, and its combination treatment. Representative images are shown from n = 3 independent experiments. (B) Colony-formation assays of uveal melanoma cells to zoledronic acid, GSK126, and its combination treatment. Representative images are shown from n = 3 independent experiments. See also Figure S4.

**Figure 5:**
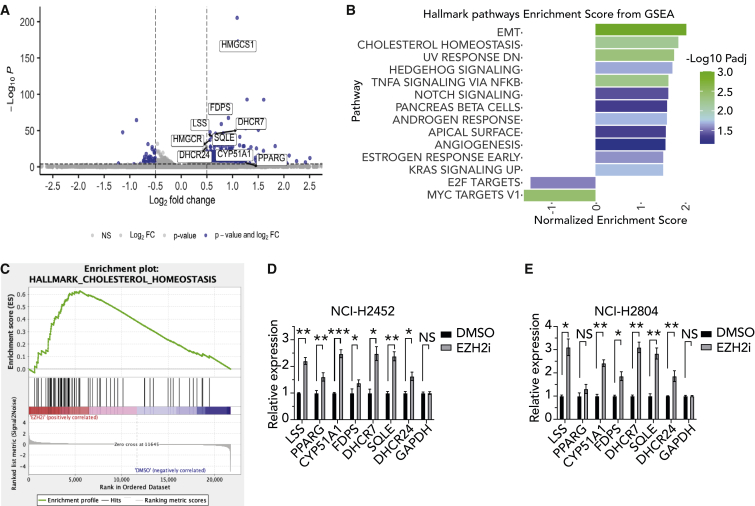
Treatment of BAP1-deficient cells *in vitro* with an EZH2 inhibitor increases expression of cholesterol homeostasis related genes (A) Volcano plot representing the changes in gene expression of the cholesterol homeostasis genes (labeled) upon EZH2 inhibition in NCI-H2731 cells. The x axis shows log2FC (treated/control), and the y axis shows the adjusted p values, which were calculated by differential expression test (using the DESeq2 package in R). A gene was considered to be differentially expressed with a p <0.0001 and a log2FC >0.5 (in blue). (B) Pathway enrichment of hallmark gene sets within the MSigDB upon EZH2 inhibition in NCI-H2731 cells; shown are the pathways with FDR <0.25. (C) GSEA plot showing enrichment cholesterol homeostasis in gene expression data of EZH2 inhibited BAP1-deficient mesothelioma cells. (D) qPCR validation of upregulation of cholesterol homeostasis genes in BAP1-deficient cell line NCI-H2452 upon 48 h treatment with EZH2 inhibitor relative to DMSO control (mean ± SD; n = 3 independent experiments). (E) Similarly for NCI-H2804 (mean ± SD; n = 3 independent experiments). p values were determined by two-tailed unpaired Student’s t test; ∗p < 0.05, ∗∗p < 0.01, and ∗∗∗p < 0.001. See also Figure S5.

Currently, there is no registered second-line therapy for patients with mesothelioma who fail first-line therapy. Moreover, despite a high expression of EGFR or VEGF in mesothelioma, none of the single-agent therapies with (multi)targeted tyrosine kinase inhibitors have been successful.^32^^,^^33^^,^^34^ Therefore, there is a need to explore new combinatorial targeted therapies. Previously, we and others have shown that EZH2 inhibition is effective in *BAP1*-deleted mesothelioma.^15^^,^^17^ Our current results confirm this and also identify a new targetable vulnerability, the mevalonate pathway. Therefore, we hypothesized that inhibiting both EZH2 and the mevalonate pathway might be an effective combination treatment strategy for *BAP1*-deleted tumors. To test this hypothesis, we have performed long-term colony-formation assays in a panel of *BAP1*-proficient and -deficient human cell lines. We observe that *BAP1*-deficient human mesothelioma cell lines (except H28) are hypersensitive to the combined treatment of EZH2 inhibitor GSK126 and mevalonate pathway inhibitor ZA (Figures 4A and S4A–S4J). We extended our pharmacological observations to another malignancy, uveal melanoma, which also shows a high frequency of mutations in *BAP1*. Like mesothelioma, only *BAP1*-deficient, and not WT, uveal melanoma cell lines are sensitive to combined treatment with EZH2 and mevalonate pathway inhibitors (Figures 4B and S4K**–**S4S). These observations clearly indicate that the sensitivity to the drug combination is strictly dependent on loss of *BAP1*. To gain insight into the observed enhanced sensitivity of the combination, we treated *BAP1* WT (H2810) and *BAP1* mutant (H2731) mesothelioma cell lines with EZH2 inhibitor GSK126 and analyzed mRNA expression profiles. Using DeSeq2 differential expression analysis, we were able to identify multiple genes belonging to cholesterol metabolism to be upregulated upon treatment with EZH2i only in cells lacking *BAP1* (Figures 5A and S5A–S5C). Notably, performing gene set enrichment analysis (GSEA) for hallmarks gene sets on our data shows a clear enrichment of the cholesterol homeostasis pathway due to EZH2 inhibition in the *BAP1*-deficient H2731 mesothelioma cell line (Figures 5B, 5C, and S5D). Further, we validated these observations in two additional *BAP1*-negative mesothelioma cell lines and consistently observed that genes belonging to cholesterol metabolism are upregulated upon treatment with an EZH2 inhibitor (Figures 5D and 5E). A recent study in head and neck cancer squamous carcinoma (HNSCC) cells showed that the inhibition of EZH2 induced genes involved in cholesterol biosynthesis such as squalene and consequently synergized with an inhibitor of squalene epoxidase.^35^ Moreover, we see that *BAP1* mutant cells are also highly sensitive to a combination of squalene epoxidase inhibitor (Terbinafine HCl) plus EZH2i (Figures S5E and S5F). In addition, the combined targeting of EZH2 and an enzyme upstream in the mevalonate pathway, i.e., HMG-coenzyme A (CoA) by lovastatin, also results in reduced survival of *BAP1-*deficient mesothelioma cells (Figures S5G and S5H). Taken together, the results indicate that EZH2 inhibition in *BAP1*-deficient cells upregulates mevalonate pathway genes. This appears to go hand in hand with an addiction to the products of this pathway resulting in an enhanced sensitivity to their inhibitors.

### EZH2 inhibition in combination with ZA limits tumor growth and prolongs survival of *Bap1*-deficient mesothelioma mice

**Figure 6:**
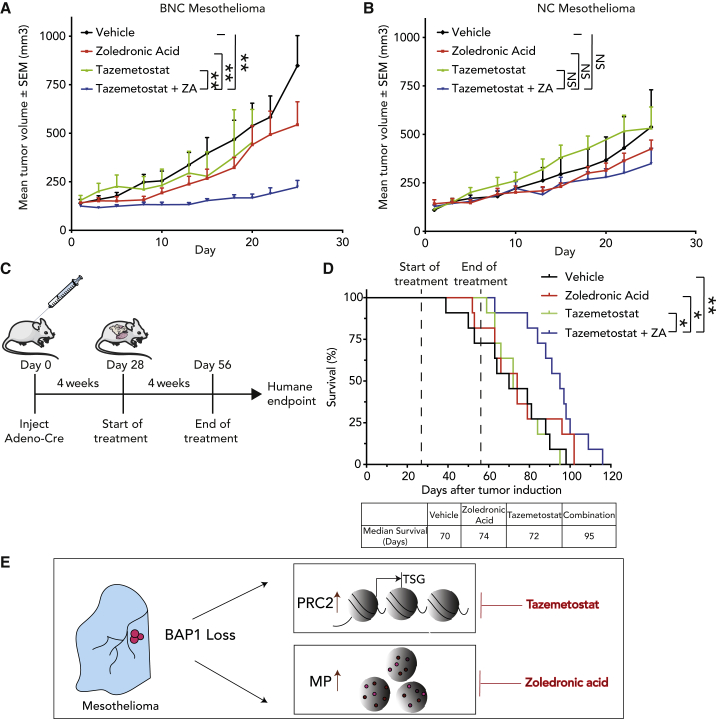
EZH2 inhibition in combination with zoledronic acid (ZA) limits tumor growth and prolongs survival of *Bap1*-deficient mesothelioma mice (A and B) NSG mice with BNC and NC xenografts were treated with vehicle, ZA (0.1 mg/kg every other day), tazemetostat (250 mg/kg daily), or a combination. Shown is mean tumor volume over time (tumor volume ± SEM; n = 8 mice per treatment group). p value was determined by two-tailed unpaired Student’s t test; ∗p < 0.05, ∗∗p < 0.01. (C) Schematic representation of experimental workflow of autochthonous *Bap1*-deficient mesothelioma mouse model. (D) Kaplan-Meier curve comparing the survival of vehicle-, ZA-, tazemetostat-, and tazemetostat plus ZA-treated mice (n = 11 mice per treatment group). ZA was administered intraperitoneally once daily at 0.2 mg/kg. Tazemetostat was administered twice daily via oral gavage at 250 mg/kg. Dashed lines indicate start and end of treatment. The table depicts the median survival of each group. p value was determined by log rank (Mantel-Cox) test; ∗p < 0.05, ∗∗p < 0.01. (E) A schematic summarizing the consequences of BAP1 loss in mesothelioma cells and their resulting sensitivities to PRC2 inhibitor (tazemetostat) and mevalonate pathway inhibitor (ZA). See also Figure S6.

To assess the efficacy of our combined drug inhibition *in vivo*, we grafted cell lines derived from *NC* and *BNC* mice in NOD-Scid IL2Rγ^null^ mice. Tumor-bearing mice were treated with tazemetostat, an EZH2 inhibitor, ZA, or a combination of both drugs, and we monitored tumor volume over time. We observed that the combination of tazemetostat and ZA resulted in significant growth inhibition of tumors in *Bap1*-deficient xenografts compared with *Bap1*-proficient xenografts, validating our *in vitro* observations (Figures 6A and 6B).

Based on these encouraging observations, we moved toward evaluating our findings in our autochthonous model of mesothelioma, which closely mimics the human malignancy. Our earlier published immuno-competent BNC mouse model rapidly develops highly aggressive mesothelioma and thus represents a worst-case scenario.^15^

Firstly, we initiated tumor development by conditional deletion of the BNC alleles and after 4 weeks started administration of tazemetostat, ZA, or the combination. The mice were monitored until they showed signs of respiratory distress and significant weight loss (humane endpoint) (Figure 6C). The combined treatment with ZA and tazemetostat significantly prolonged the median survival by approximately (approx.) 4 weeks (95 days) compared with vehicle control (70 days) (Figure 6D). Treatment with tazemetostat and ZA at a concentration of 250 mg/kg twice daily and 0.2 mg/kg once daily, respectively, provided limited benefit when used as a single agent (72 and 74 days, respectively). Also, monitoring body weight during combination treatment showed no differences compared with vehicle treatment at these doses, making this combination suitable for future dose-escalation studies (Figures S6A and S6B). Importantly, compared with other *in vivo* studies the drug dosage (concentration and/or frequency) of tazemetostat and ZA used here were below what was clinical used.^17^^,^^36^^,^^37^^,^^38^^,^^39^

Taken together, our observations demonstrate that loss of BAP1 results in deregulation of epigenetic and metabolic pathways. Therefore, simultaneous targeting of these two crucial pathways (PRC2 inhibition plus mevalonate pathway inhibition) might be an attractive strategy for treating *BAP1*-deficient mesothelioma (Figure 6E). As both drugs are already used in the clinic, the proposed combination therapy can be evaluated for its efficacy and safety in phase I/II studies.

## Discussion

The current standard of care for mesothelioma includes newly approved immune checkpoint blockade (ICB) therapies.^2^ Although ICB therapies do improve overall survival for patients with mesothelioma compared with chemotherapeutic agents, there is still much need for improvement. Identifying biomarker-based dependencies and exploiting them have a high potential to lead to new treatment options. As reported for other solid tumors, stratification of patients based on biomarkers could add much needed therapeutic strategies against this highly aggressive disease.^40^^,^^41^^,^^42^^,^^43^

In the current study, we have used focused CRISPR-genetic screens and mouse mesothelioma cell lines with a defined genetic background for identifying targetable vulnerabilities specifically associated with *Bap1* loss. This tumor suppressor could serve as a potential biomarker as it is mutated in a significant number of patients with mesothelioma. We show that *BAP1*-deficient mesothelioma is synthetically lethal to mevalonate pathway inhibition and that it is possible to pharmacologically exploit this lethality by repurposing ZA, a drug routinely used in clinic.^24^^,^^25^^,^^26^^,^^27^ Previously conducted clinical trials using ZA show a modest benefit in patients with mesothelioma. However, these trials did not stratify patients on the basis of potential biomarkers; based on our results, we propose that the use of BAP1 as a biomarker could increase the efficacy of ZA in mesothelioma.

We also have described epigenetic and expression changes that are exclusively associated with *BAP1* deficiency and its potential implications in mesothelioma progression. Lately, there have been reports describing precise changes in Polycomb proteins, uncovering related vulnerabilities that can be targeted in *BAP1*-loss-associated malignancies.^29^^,^^30^ However, in mesothelioma, the H2AK119ub1 does not seem to primarily influence the expression of genes when co-occupied with H3K27me3. Our results in *BAP1*-deficient mouse and human mesothelioma indicate that it is the increased PRC2 occupancy at key promoters that influences the expression status of target genes. Moreover, this elevated PRC2 occupancy in BAP1-altered mesothelioma creates selective sensitivity to PRC2 inhibitors. However, our results from mouse models show that tazemetostat may show limited efficacy when used as a single agent. A recently published clinical study corroborates our findings where patients with mesothelioma with *BAP1* deficiency derived limited clinical benefits from tazemetostat treatment.^44^ More in general, monotherapies often have limited efficacy and quickly result in drug resistance.^45^^,^^46^^,^^47^

Here, we show that combined targeting of mevalonate pathway and PRC2 dependency convey significant survival benefit in aggressive mouse models of mesothelioma, and thus this combination might be a more viable option for treating *BAP1-*deficient mesothelioma. In addition, we demonstrate that the mevalonate pathway and PRC2 vulnerabilities extend to other *BAP1*-loss-associated malignancies such as uveal melanoma, in which over 95% of metastases have lost *BAP1*.^6^

Recently, a study by Xu et al. in HNSCCs showed a clear synergy between the combined targeting of the cholesterol pathway (downstream of mevalonate pathway) with PRC2 inhibition.^35^ This is in line with our observations that EZH2 inhibition upregulates genes of the cholesterol and mevalonate biosynthesis pathways in *BAP1*-mutated settings with concomitant addiction to these pathways. Additionally, previously conducted studies in mice too have demonstrated a direct link between BAP1 loss and the deregulation of the cholesterol/mevalonate pathways.^23^ These observations clearly indicate that BAP1-deficient mesothelioma gains dependency on both PRC2 and mevalonate/cholesterol pathways. Together with the availability of inhibitors already used in clinic against PRC2 (tazemetostat) and mevalonate pathway (ZA), the observed enhanced sensitivity *in vitro* makes this a very attractive combination.

Overall, our study illustrates the potential of a combination therapy targeting two key pathways in *BAP1*-deficient malignancies and thereby adding a new therapeutic option to the treatment landscape of mesothelioma.

### Limitations of the study

Although we have extensively validated the tolerability and efficacy of the EZH2i plus mevalonate inhibition combination in preclinical models, the results may vary regarding these drugs’ tolerability, efficacy and pharmacokinetics when testing the combination in patients. We lack experimental quantification of the ChIP sequencing (ChIP-seq) signal due to the non-availability of foreign spikein chromatin. Future work needs to identify concrete mechanisms of how the BAP1-deficient cells are sensitive to mevalonate pathway inhibition.

## STAR★Methods

### Key resources table

**Table:** 

REAGENT or RESOURCE	SOURCE	IDENTIFIER
**Antibodies**		

BAP1 (D7W7O) Rabbit mAb	Cell Signaling Technology	Cat# 13271; RRID:AB_2798168
RAP1A (C-10) Mouse mAb	Santa Cruz Biotechnology	Cat# sc-373968; RRID:AB_10917062
Tri-Methyl-Histone H3 (Lys27) (C36B11) Rabbit mAb	Cell Signaling Technology	Cat# 9733, RRID:AB_2616029
a-Tubulin Mouse mAb	Sigma-Aldrich	Cat# T9026, RRID:AB_477593
Goat anti-Rabbit IgG (H + L) Cross-Adsorbed Secondary Antibody, HRP	Thermo Fisher Scientific	Cat# G-21234, RRID:AB_2536530
Goat anti-Mouse IgG (H + L) Secondary Antibody, HRP	Thermo Fisher Scientific	Cat# 62-6520, RRID:AB_2533947

**Bacterial and virus strains**		

Mouse Kinome CRISPR Knockout Library (Brie)	Doench et al.^21^	Addgene #75316
FH1-tUTG	Aubrey et al.^48^	Addgene #70183
pLKO5.sgRNA.EFS.GFP	Heckl et al.^49^	Addgene #57822

**Chemicals, peptides, and recombinant proteins**		

Zoledronic Acid	Medkoo Biosciences	100950 CAS: 165800-06-6
GSK126	Selleck Chemicals	S7061 CAS: 1346574-57-9
Tazemetostat	Medkoo Biosciences	406265 CAS: 1403254-99-8
Lovastatin (MK-803)	Selleck Chemicals	S2061 CAS: 75330-75-5
Terbinafine HCl (KWD 201)	Selleck Chemicals	S2557 CAS: 78628-80-5

**Critical commercial assays**		

ReliaPrep™ RNA Miniprep Systems	Promega	Cat# Z6012
Tetro cDNA synthesis kit	Meridian Bioscience	Cat# BIO-65043

**Deposited data**		

RNA sequencing data	This paper	GSE222376
CRISPR/CAS9-screen MAGeCK results	This paper	Data available upon request
Patient survival and expression data (upon consent)	Genentech	EGAS00001001563
ChIP-seq data	This paper	GSE145022

**Experimental models: Cell lines**		

Human: NCI-H226	Wellcome Trust Sanger Institute	RRID:CVCL_1544
Human: NCI-H226 + BAP1 WT	Wellcome Trust Sanger Institute	N/A
Human: NCI-H2452	Wellcome Trust Sanger Institute	RRID:CVCL_1553
Human: NCI-H2731	Wellcome Trust Sanger Institute	RRID:CVCL_U995
Human: NCI-H28	Wellcome Trust Sanger Institute	RRID:CVCL_1555
Human: NCI-H2804	Wellcome Trust Sanger Institute	RRID:CVCL_U998
Human: NCI-H2810	Wellcome Trust Sanger Institute	RRID:CVCL_U999
Human: NCI-H2818	Wellcome Trust Sanger Institute	RRID:CVCL_V000
Human: MSTO-211H	ATCC	RRID:CVCL_1430
Human: MPP-89	Wellcome Trust Sanger Institute	RRID:CVCL_1427
Human: MP38	Wellcome Trust Sanger Institute	RRID:CVCL_4D11
Human: MP41	Wellcome Trust Sanger Institute	RRID:CVCL_4D12
Human: MP46	Wellcome Trust Sanger Institute	RRID:CVCL_4D13
Human: Mel270	Wellcome Trust Sanger Institute	RRID:CVCL_C302
Human: MM28	Wellcome Trust Sanger Institute	RRID:CVCL_4D15
Human: MM66	Wellcome Trust Sanger Institute	RRID:CVCL_4D17
Human: OMM.1	Wellcome Trust Sanger Institute	RRID:CVCL_6939
Human: 92.1	Wellcome Trust Sanger Institute	RRID:CVCL_8607
Mouse: NC	Badhai et al.^15^	N/A
Mouse: BNC	Badhai et al.^15^	N/A
Mouse: BNCP	Badhai et al.^15^	N/A

**Experimental models: Organisms/strains**		

Mouse: NOD-Scid IL2Rγnull	Jackson Laboratory	RRID:IMSR_JAX:005557
Mouse: Autochthonous Bap1-deficient mesothelioma model	Badhai et al.^15^	N/A

**Oligonucleotides**		

sgRNA targeting sequence: *Mvk:* CACCGCAAGGTCCCGCGGAGTACCA	This paper	N/A
sgRNA targeting sequence: *Pmvk:* CACCGCTCTCTGGTCCACTCAAGG	This paper	N/A
Inducible shRNA targeting sequence: *BAP1*: GAGUUCAUCUGCACCUUUA	This paper	N/A
(ChIP-)qPCR primers see Table S1	This paper	N/A

**Recombinant DNA**		

**Software and algorithms**		

GraphPad Prism v.9	GraphpadSoftware	https://www.graphpad.com:443/
FlowJo v.10.6.0	FlowJo, LLC	https://www.flowjo.com/
R (v4.1.1)	R	https://cran.r-project.org/
RStudio (v1.4.1106)	RStudio, PBC	https://www.rstudio.com/
DESeq2 (version1.30.1)	Love et al.^50^	https://bioconductor.org/packages/release/bioc/html/DESeq2.html
MAGeCK algorithm (v0.5.9.4)	Li et al.^51^	https://sourceforge.net/p/mageck/wiki/
GSEA (v4.1.0)	UC San Diego	https://www.gsea-msigdb.org/gsea/index.jsp

### Resource availability

#### Lead contact

Further information and requests for resources and reagents should be directed to and will be fulfilled by the lead contact, Maarten van Lohuizen (m.v.lohuizen@nki.nl).

#### Materials availability

This study did not generate new, unique reagents.

### Experimental model and subject details

#### Cells

Mouse mesothelioma cell lines were previously generated in our laboratory and cultured in Dulbecco’s Modified Eagle Medium/Nutrient Mixture F-12 (DMEM/F12 + Glutamax; Gibco), supplemented with 4 μg/mL Hydrocortisone (Sigma), 5 ng/ml murine EFG (Sigma), insulin-transferrin-selenium solution (ITS; Gibco), 10% fetal calf serum (FCS; Capricorn), and 1% penicillin and streptomycin (Gibco).^15^^,^^52^ Human mesothelioma cell lines were obtained from the American Type Culture Collection (ATCC) and were cultured in mammalian cell culture medium as specified above. Uveal melanoma cell lines, also obtained from ATCC, were cultured in either RPMI 1640 (RPMI-1640; Gibco) or Dulbecco’s Modified Eagle Medium (DMEM; Gibco) supplemented with 10% or 20% FCS and 1% penicillin/streptomycin. All cell lines were maintained at 37°C in a humidified atmosphere containing 5% carbon dioxide (CO_2_) and were tested for mycoplasma contamination using MycoAlert Mycoplasma detection kit (Lonza). The human cell lines were authenticated using short tandem repeat STR DNA profiling.

#### Animal studies

All animal procedures were performed in accordance with Dutch law and the institutional committees (Animal experimental committee and Animal welfare body) overseeing animal experiments at the Netherlands Cancer Institute, Amsterdam. Mice were housed under standard feeding, light cycles, and temperature with *ad libitum* access to food and water. All mice were housed in disposable cages in the laboratory animal center (LAC) of the NKI, minimizing the risk of cross-infection, improving ergonomics and obviating the need for a robotics infrastructure for cage-washing. The mice were kept under specific pathogen free (SPF) conditions.

To establish xenografts, 5 × 10^6^ mouse mesothelioma derived cells in 100μL PBS with 50% Matrigel (Corning) were subcutaneously implanted into the flank of 6–10 weeks old NOD-Scid IL2Rγnull (NSG) mice (Jackson Laboratory). Tumor growth was monitored by slide caliper 3 times a week (volume = length x width^2^/2). Tumors were allowed to grow to ∼150 mm^3^ in size before randomization into control and treatment groups. Mice were treated for 28 days. Zoledronic Acid was administered intraperitoneally every alternate day at 0.1 mg/kg. Tazemetostat was administered twice daily via oral gavage at 250 mg/kg. Mouse body weight was monitored every day.

Mice and induction of autochthonous mesothelioma were executed as described previously.^15^ Treatments were executed by two independent members of the Intervention Unit of the Netherlands Cancer Institute. Tumor measurements and health assessments of mice were performed in a blinded manner. Male and female mice were equally distributed over treatment groups with a similar mean weight in each group. Mice receiving different therapies were allowed to be housed in the same cage. Treatment started 4 weeks after tumor induction and continued for 28 days. ZA was administered intraperitoneally once daily at 0.2 mg/kg. Tazemetostat was administered twice daily via oral gavage at 250 mg/kg. Mice were monitored daily for weight loss and breathing difficulties. Mice were sacrificed upon signs of illness (breathing abnormalities, kyphosis, weight loss). Kaplan-Meier curves were generated at the end of the experiment.

### Method details

#### Kinome-centered CRISPR-Cas9 drop-out screen

*Bap1*-deficient (*BNC, BNCP*) and –proficient (*NC*) mesothelioma cells were transduced with lentiviral particles containing the mouse Brie kinome pooled library (AddGene, 75,316) at low M.O.I. (∼0.3) for single viral integration and a representation of 500-fold in the selected population. Cells were selected with puromycin for 3 days (=T_0_) and maintained at a 500x coverage for 2 weeks (=T_1_). The abundance of each gRNA was determined by PCR recovery followed by Illumina deep sequencing. For sequence depth normalization, a relative total size factor was calculated for each sample, by dividing the total counts of each sample by the geometric mean of all totals. All the values of a sample were then divided by the sample size factor. The normalized data was analyzed in the following way; first a differential analysis on the sgRNA level between two conditions was done with DESeq2, producing a log2 fold change value, a p value and its own test statistic. The results of this analysis were sorted on the DESeq2 test statistic putting the most depleted sgRNA at the top. MAGeCK’s RRA tool was used to analyze each gene for enrichment of the sgRNAs toward the top.^51^ The p value of this enrichment analysis was then corrected for multiple testing, using the Benjamini-Hochberg method. For each gene the median log2 fold change was calculated over its sgRNAs. Hits were selected based on the following criteria: the genes that had an FDR ≤0.1 and a median log2 fold change ≤ to −1. For BNCP line the log2 fold change threshold was set to −0.4.

#### Generation of knockdown and knock-out cell lines

For *BAP1* knockdown experiments, we used doxycycline-inducible FH1-tUTG-RNAi vectors (Taconic Artemis) targeting the following sequence: 5′- GAGUUCAUCUGCACCUUUA-3′’.^48^^,^^53^^,^^54^ HEK293t cells in 10cm plates were transduced using 3.5 μg of FH1-tUTG-*BAP1*, 1.1 μg VSV-G, 0.8 μg REV, and 1.6 μg POL. Virus was harvested and used to infect human mesothelioma cell lines. GFP positive cells were sorted by flow cytometry.

For Cas9-induced knockout, we used Alt-R CRISPR Guide RNAs (IDT DNA). crRNA and tracrRNA were mixed in equimolar concentrations to create a final duplex concentration of 3μM. The duplex was mixed with an equimolar amount of Alt-R spCas9 enzyme to form an RNP complex. The RNP complex was reverse transfected into cells using Lipofectamine RNAiMax (Invitrogen, product #13778075).

#### Western blot analysis

Whole-cell pellets were lysed in RIPA buffer (50 mM Tris, pH 8.0, 50 mM NaCl, 1.0% NP-40, 0.5% sodium deoxycholate, and 0.1% SDS) containing protease inhibitor cocktail (Complete; Roche) and phosphate inhibitors (10 mM NaF final concentration, 1 mM Na_3_VO_4_ final concentration, 25mM β-Glycerophosphate final concentration, 1mM PMSF, and 1 mM Na_4_P_2_O_7_ final concentration). Protein concentrations were measured using Protein Assay Dye reagent (Bio-rad) and a Nanodrop 2000c machine. Equal amounts of protein were loaded onto 4–12% Bis-Tris gels (NuPage-Novex, Invitrogen) and transferred onto nitrocellulose membranes (0.2 μm; Whatman). Membranes were blocked in 5% BSA in PBS (PBS) with 0.1% Tween 20 (PBST) for 1 h, incubated with primary antibodies in PBST 1% BSA overnight at 4°C, and incubated with secondary antibodies coupled to HRP for 45 min in PBST 1% BSA at room temperature. Antibody detection was accomplished using Amersham ECL detection reagent (GE healthcare). Membranes were imaged on a BioRad ChemiDoc XRS+. The following antibodies were used for western blot analyses: BAP1 D7W70 (Cell Signaling, 13271S), RAP1A C-10 (Santa Cruz Biotechnology, sc-373968), Tri-Methyl-Histone H3 (Lys27) C36B11 (Cell Signaling, 9733S), anti-Tubulin (Sigma, T9026).

#### RNA isolation and RT-qPCR

Total RNA was extracted from cells using ReliaPrep (Promega). Reverse transcription was performed with the Tetro cDNA synthesis kit (Meridian) using Random Hexamers. qPCR was performed with Power SYBR green master mix (Applied Biosystems) in triplicates using the QuantStudio 5 Real-Time PCR System (ThermoFisher). Data were normalized against GAPDH. Primers used are listed in Table Methods S1.

#### Cell viability assays

Prior to cell viability assays optimal seeding density of cell lines was derived from growth curves. Cells were counted with HyClone Trypan Blue (Cytiva) using a TC20 automated cell counter (Bio-Rad) and alive cells were seeded into 384-well plates in 50μL of culture medium. Drug compounds, DMSO negative control, or PAO positive control was added after 24 h using the D300e digital dispenser (TECAN) and cells were grown for 72 h. Subsequently, cells were incubated for 4 h with Resazurin (Sigma) and plates were read using an Infinite M1000 pro plate reader (TECAN).

#### Colony-formation assays

Again, prior to colony formation assay optimal seeding densities were determined. Cells were seeded in 6-well culture plates and allowed to adhere overnight. Cells were then cultured in the continuous presence of drug compound(s) or DMSO. After 10 days plates were fixed using 4% Paraformaldehyde (Merck) and stained with 0.1% crystal violet solution (Sigma) in PBS with 10% EtOH. Plates were digitized on an image scanner and analyzed using the ImageJ plugin ‘ColonyArea’ as published by C. Guzman et al.^55^ Representative images of three independent experiments are shown.

#### ChIP seq analysis

ChIP-seq data used was as originally published in Badhai et al*.*^15^ Reads were aligned to mm10 using BWA, filtered for quality score ≥20, duplicate reads were removed using PICARD. Intergenic sites were defined as all genomic loci subtracted from gene bodies and backlisted regions.^56^ Promoter regions were defined as TSS +/− 5kb. Sequencing reads for relevant ChIP-seq experiments were quantified with bedtools. Counts were RPKM normalized and visualized as boxplots with R, using ggplot2, restricting y axes upper limit to 99% of normalized value for visualization purposes.

#### ChIP-qPCR

Cell lines were cross-linked with 1% methanol-free formaldehyde in PBS for 10 min Cross-linking was blocked with glycine and lysed in ChIP lysis buffer (1% SDS, 10mM EDTA, 50mM Tris-HCl). Chromatin was fragmented (200-500bp) using a Bioruptor Pico sonication device (Diagenode). Sonicated cell lysates were diluted in ice-cold ChIP dilution buffer (0.01% SDS, 1% Triton X-100, 1.2mM EDTA, 17.6mM Tris-HCl, 167mM NaCl). 20μg of sample was used per immunoprecipitation reaction, 10% was taken as input sample. Fragmented chromatin was incubated overnight at 4°C with 5μg of anti-H3K27me3 antibody (Cell Signaling, 9733S) and isotype control (IgG). The next day, samples were incubated with protein A beads (Invitrogen) for 4 h at 4°C. Chromatin-Antibody-Beads complex was then washed with once Low salt buffer (0.1% SDS, 1% Triton X-100, 2mM EDTA, 20mM Tris-HCl, 150nM NaCl), once High salt buffer (0.1% SDS, 1% Triton X-100, 2mM EDTA, 20mM Tris-HCl, 500nM NaCl), once LiCl buffer (0.25M LiCl, 1% IGEPAL-CH 630, 1% deoxycholic acid, 1mM EDTA, 10mM Tris-HCl), and twice TE buffer (1mM EDTA, 10mM Tris-HCl). Complexes were reverse cross-linked and eluded by heating with 2 volumes of Elution buffer (1% SDS, 100mM NaHCO_3_, 50mM Tris-HCl) and 5M NaCl. RNA and Proteins were removed by treatment with RNaseA and Proteinase K, DNA was removed using AMPure bead isolation (Beckman Coulter). The enrichment of immunoprecipitated DNA with anti-H3K27me3 antibody was calculated in the following way: Calculate average Ct of triplicates, calculate adjusted Ct value for input sample (Ct_10% Input_-Log(10,2)), calculate ΔCt value over input sample, calculate the percent input (100∗2^(ΔCt)). Primers used are listed in Table Methods S1.

#### RNA sequencing, analysis, and GSEA

Cells were lysed in RLT buffer (Qiagen). RNA extraction, library preparation, sequencing, and reads processing were performed by the Genomics Core Facility at the Netherlands Cancer Institute. Sequencing was performed using the Illumina HiSeq 2500 platform according to the standard procedures. RNA-sequencing reads from mouse material were aligned to the mm10 genome with hisat2, transcript quantification was performed with HTSeq. Human samples were aligned to GRCh38 and read counts per gene using gensum. Genes were annotated using Ensembl GRCh38.102. Subsequent data analyses were performed using R and Bioconductor. DESeq2 package was used for the analysis of differential gene expression in RNA sequencing samples of both mouse and human experiments.^50^ DESeq2 was used as well to retrieve log_2_-transformed, normalized transcript counts, which were subsequently used for visualization purposes. For integration with our mouse ChIP-seq data we retrieved ChIP-seq signals by determining sequencing reads at mouse promoter regions (TSS +/− 2.5 kb) of genes with bedtools and subsequent normalization and log2-normalisation of counts with DESeq2. To match gene expression changes in human mesothelioma cell lines with significant genes from the BNC vs. NC mouse model, we used the biomaRt package in R to find human orthologs of relevant genes in mice. We selected genes specifically that were significantly differentially expressed in the mouse model, and showed matching dynamics at ChIP-seq level as well as in matching dynamics in the human shRNA data. Furthermore, we selected promoters with the top 10% largest increase or decrease in H2AK119Ub1 or H3K27Me3 signal, and visualized the relative enrichment of these histone marks along with relative expression of the respective genes. Relative ChIP-signals at promoters of significant genes, and relative expression levels were visualized as row-means-subtracted values. Gene set enrichment analysis (GSEA) was performed on the differentially expressed genes using the H (hallmark) dataset from the MsigDB.^57^ The metric for ranking genes was set to Signal2Noise, all other parameters were as standard. Plots were generated using the Normalized Enrichment Score and the nominal p value.

#### Patient survival analysis

Overall survival and gene expression data was obtained from Genentech.^4^ Survival analyses were performed using the Survival package in R. Visualization was done using the Survminer package.

#### Flow cytometry analysis

For sgRNA validation we used pLKO5.sgRNA.EFS.GFP vector (a gift from Benjamin Ebert, Addgene plasmid #57822) targeting the following sequences; (*Pmvk*) 5′-CACCGCTCTCTGGTCCACTCAAGG-3′ and (*Mvk*) 5′-CACCGCAAGGTCCCGCGGAGTACCA-3′.^49^ HEK293t cells in 10cm plates were transduced using 3.5 μg of pLKO5.sgRNA.EFS.GFP, 1.1 μg VSV-G, 0.8 μg REV, and 1.6 μg POL. Virus was harvested and used to infect Cas9-positive (neomycin selected) mouse mesothelioma cell lines. Cloned single-cell suspensions were prepared by filtering cells through 35 μm cell strainers. Samples were analyzed for GFP signal using a BD LSR II SORP Flow Cytometer. Data analysis was performed using FlowJo v.10.6.0 (TreeStar).

### Quantification and statistical analysis

All statistical tests were performed using GraphPad Prism v.9 and R. Statistical significance was denoted as ∗p < 0.05, ∗∗p < 0.01, ∗∗∗p < 0.001, and ∗∗∗∗p < 0.0001. The number of independent experiments, samples, and type of statistical test are indicated in the figure legends. No statistical method was used to predetermine the sample size. *In vivo* data were compared by multiple unpaired two-sided Student’s *t* test when data were normally distributed. Survival analyses were performed by Log Rank Mantel-Cox test.

## Data Availability

This study did not generate any novel code. Raw data were deposited in the gene expression omnibus under accession number: GSE145022. All the other data used for this study are available from the lead contact upon request.

## Supplemental information

Document S1. Figures S1–S6 and Table S1

Document S2. Article plus supplemental information
