# Supplementary material for: Genetic screens reveal new targetable vulnerabilities in BAP1-deficient mesothelioma
Source: Cell Rep Med. 2023 Jan 18;4(2):100915. doi: 10.1016/j.xcrm.2022.100915 (PMC9975229; doi:10.1016/j.xcrm.2022.100915)
Supplement: Document S1. Figures S1–S6 and Table S1 [file mmc1.pdf]

Cell Reports Medicine, Volume *4*

Supplemental information

Genetic screens reveal new targetable

vulnerabilities in BAP1-deficient mesothelioma

Gaurav Kumar Pandey, Nick Landman, Hannah K. Neikes, Danielle Hulsman, Cor Lieftink, Roderick Beijersbergen, Krishna Kalyan Kolluri, Sam M. Janes, Michiel Vermeulen, Jitendra Badhai, and Maarten van Lohuizen

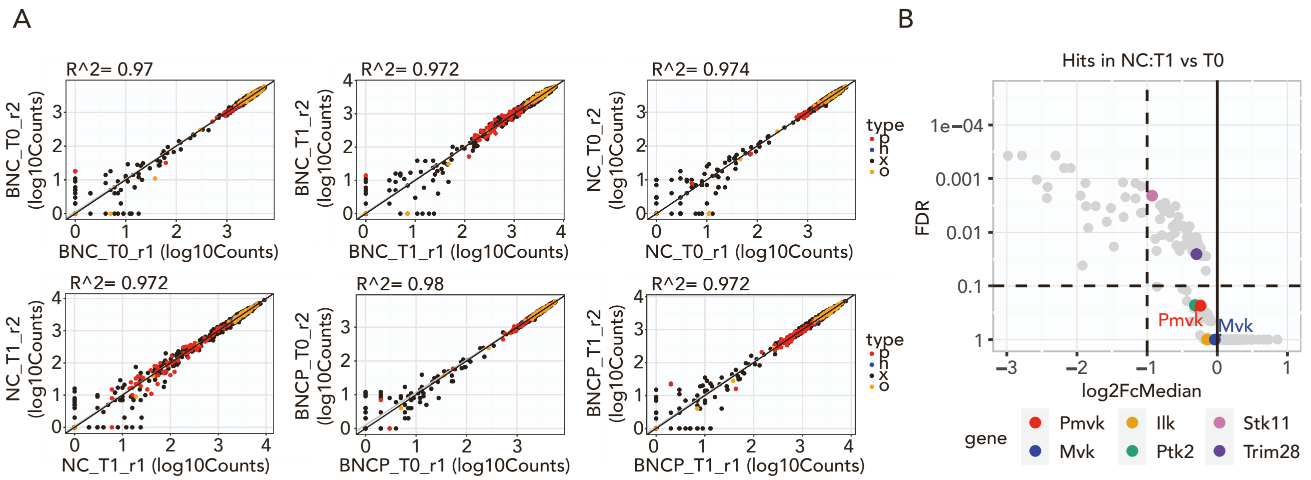

Supplementary Figure S1. CRISPR-Cas9 kinome screen in mouse mesothelioma cells with or without BAP1, related to Figure 1

(A) Plots showing the correlation between the replicates of the different samples.
(B) Volcano plot showing that the significant hits are not dropped-out in NC cells comparing T1 vs T0 (FDR≤0.1, Log2FC≤-1).

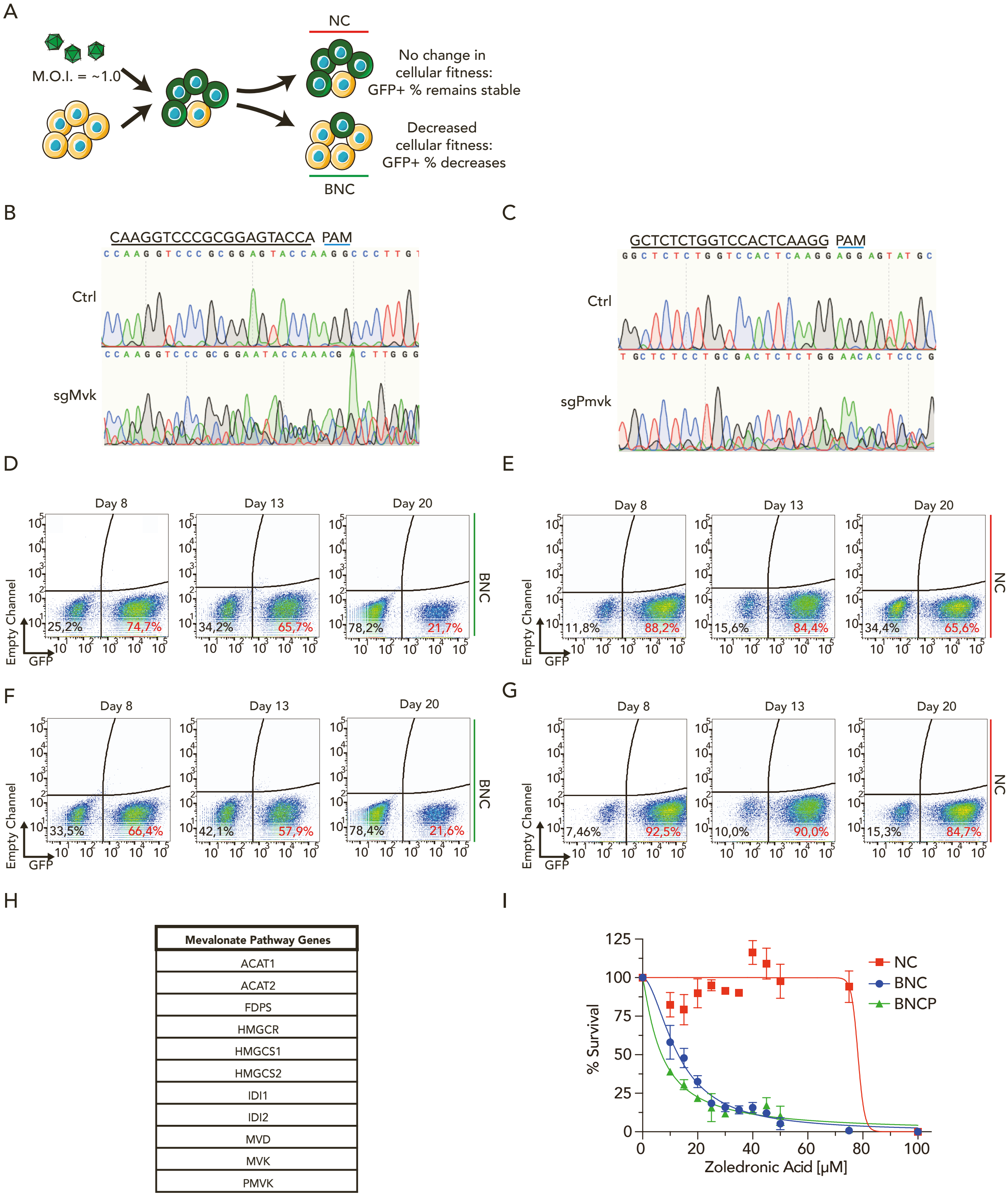

Mevalonate Pathway Genes
ACAT1
ACAT2
FDPS
HMGCR
HMGCS1
HMGCS2
IDI1
IDI2
MVD
MVK
PMVK

Supplementary Figure S2. Bap1-deficient mouse mesothelioma cell fitness is decreased upon depletion of essential mevalonate pathway genes MVK and PMVK, related to Figure 2

(A) Schematic representation of the experimental lay-out and results. **(B)** Snapshot of DNA sequencing results in SnapGene showing the control sequence and the sequence of the cell line transfected with sgMvk. Sequence of guide RNA is indicated in black and PAM sequence in blue. **(C)** Likewise for cells transfected with sgPmvk. **(D-E)** Flow cytometry plots showing the percentages of GFP+ cells in BAP1-deficient mouse mesothelioma cell line with sgPMVK:GFP over time. The x-axis represents the expression of GFP, the y-axis is an empty channel (D), likewise for BAP1-proficient mouse mesothelioma cell line (E). **(F-G)** Flow cytometry plots showing the percentages of GFP+ cells in BAP1-deficient mouse mesothelioma cell line with sgMVK:GFP over time. The x-axis represents the expression of GFP, the y-axis is an empty channel (F), likewise for BAP1-proficient mouse mesothelioma cell line (G). **(H)** List of the gene names that were used in patient survival analysis based on the expression of mevalonate pathway genes. **(I)** IC50 curves of Zoledronic Acid in mouse Mesothelioma cell lines showing a shift in sensitivity upon absence of Bap1.

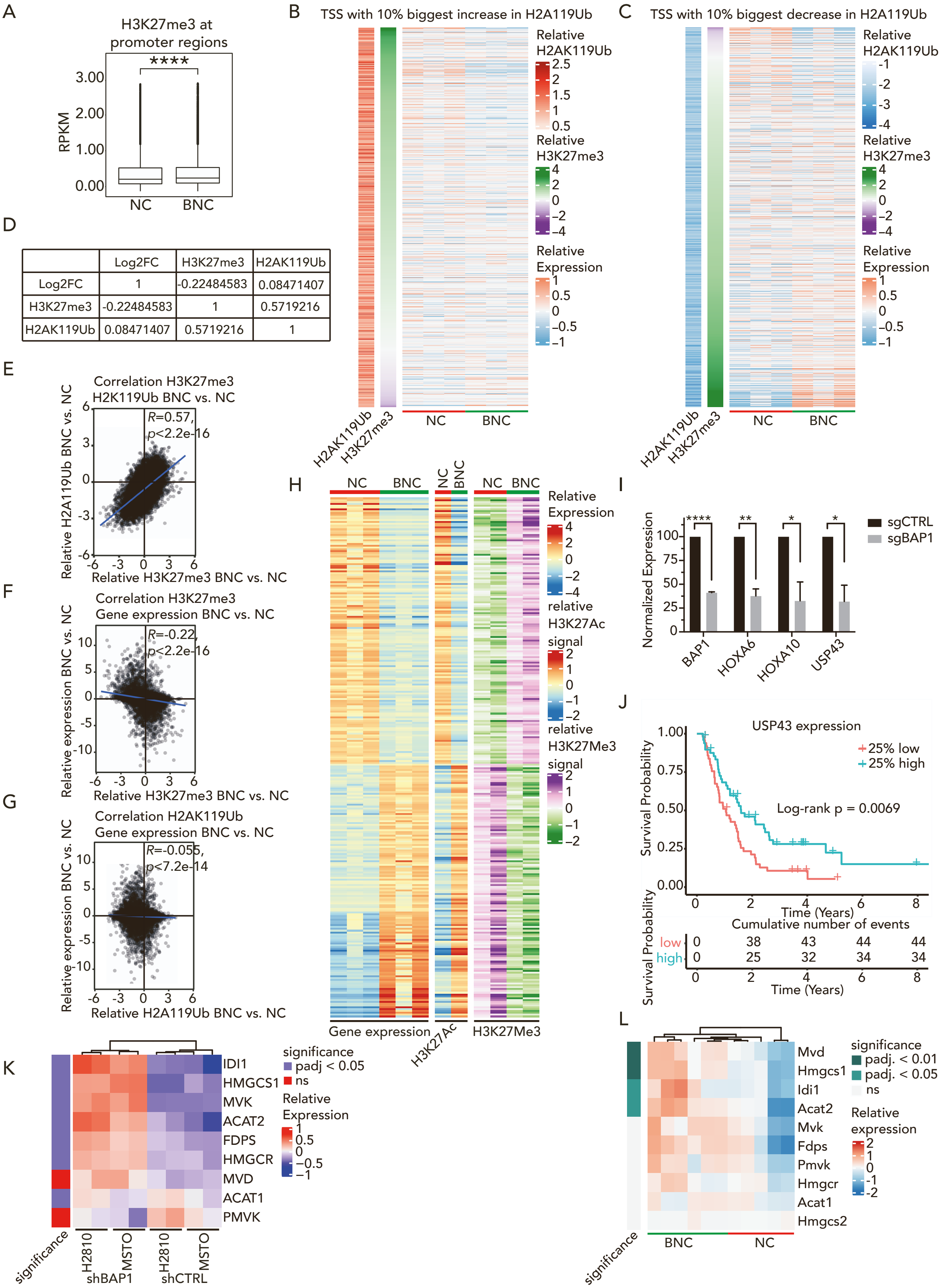

	Log2FC	H3K27me3	H2AK119Ub
Log2FC	1	-0.22484583	0.08471407
H3K27me3	-0.22484583	1	0.5719216
H2AK119Ub	0.08471407	0.5719216	1

Supplementary Figure S3. Elevated PRC2-mediated H3K27me3 occupancy silences gene expression upon BAP1 loss in mouse and human mesothelioma, related to Figure 3

(A) Boxplots representing ChIP-seq RPKM levels in the NC and BNC cells at promoter regions for H3K27me3, showing a significant increase in the relative levels of H3K27me3 in BNC cells.
(B) Heatmap representing gene expression, as well as changes in H3K27me3 and H2AK119ub ChIP signal at the top 10 percent of genes with the biggest increase in H2AK119ub1 occupation at TSS +/- 5kb between NC and BNC cells (n = 3, independent samples per group).
(C) Heatmap representing gene expression, as well as changes in H3K27me3 and H2AK119ub ChIP signal at the top 10 percent of genes with the biggest decrease in H2AK119ub1 occupation at TSS +/- 5kb between NC and BNC cells (n = 3, independent samples per group).
(D) Matrix of partial correlation analysis results for chromatin marks H3K27me3 and H2AK119Ub and Log2 fold change of gene expression.
(E) Scatterplot showing the positive correlation between the chromatin marks.
(F) Scatterplot showing an anti-correlation between relative H3K27me3 levels and gene expression.
(G) Scatterplot between relative H2AK119Ub levels and gene expression.
(H) Heatmap of genes (n = 285) that are differentially expressed between BAP1 proficient and deficient mouse mesothelioma (n = 3, biologically independent samples per group) with corresponding chromatin changes in H3K27me3 and H3K27Ac mark.
(I) qPCR measurement of BAP1, HOXA6, HOXA10 and USP43 upon sgRNA knockout of BAP1 relative to sgRNA control in H2810 mesothelioma cells (mean ± s.d.; n = 3 independent experiments). P values were determined by two-tailed unpaired Student's t-test; *P < 0.05, **P < 0.01, ***P < 0.001, and ****P < 0.000D.
(J) Kaplan-Meier curve indicating overall survival (OS) of patients (n = 197) with mesotheliomas and expression of USP43. Top quartile (25% of patients with highest expression) of USP43 expression versus bottom quartile (25% of patients with lowest expression). The graph depicts p-value obtained using the Log-rank test.
(K) Heatmap of relative expression of genes in the mevalonate pathway for human mesothelioma cell lines with inducible shBAP1 construct.
(L) Heatmap of relative expression of genes in the mevalonate pathway between Bap1 proficient and deficient mouse mesothelioma cell lines.

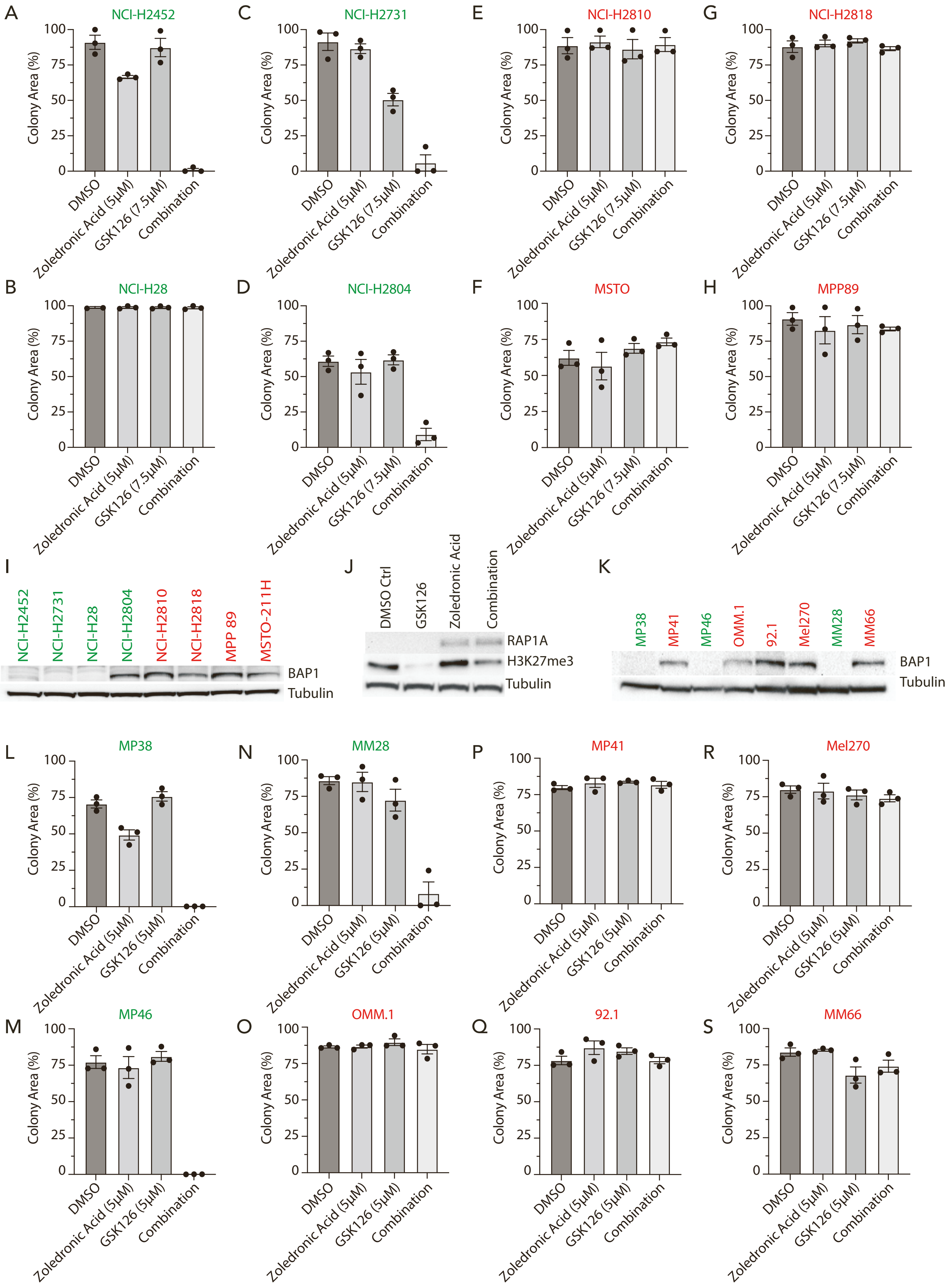
A
NCI-H2452
C
NCI-H2731
E
NCI-H2810
G
NCI-H2818
B
NCI-H28
D
NCI-H2804
F
MSTO
H
MPP89
Colony Area (%)
100
75
50
25
0
DMSO
Zoledronic Acid (5μM)
GSK126 (7.5μM)
Combination
I
NCI-H2452
NCI-H2731
NCI-H28
NCI-H2804
NCI-H2810
NCI-H2818
MPP 89
MSTO-211H
BAP1
Tubulin
J
DMSO Ctrl
GSK126
Zoledronic Acid
Combination
RAP1A
H3K27me3
Tubulin
K
MP38
MP41
MP46
OMM.1
92.1
Mel270
MM28
MM66
BAP1
Tubulin
L
MP38
N
MM28
P
MP41
R
Mel270
M
MP46
O
OMM.1
Q
92.1
S
MM66
Colony Area (%)
DMSO
Zoledronic Acid (5μM)
GSK126 (5μM)
Combination

Supplementary Figure S4: Quantification of BAP1-deficient and proficient human mesothelioma and uveal melanoma cell lines, related to Figure 4

(A-H) The quantifications of three independent human mesothelioma clonogenicity assays as shown in Figure 4A. Data are mean±S.E.M., n = 3 independent experiments. **(I)** Western blot showing the BAP1 protein status (green indicates mutated genotype, red indicates wild-type) of the human mesothelioma cell lines used for long-term clonogenicity assays. NCI-H2804 is a catalytically inactive mutant and therefore shows a band on Western blot. **(J)** Western blot showing intended inhibitory effects of inhibitors used in clonogenicity assays. Effect of Zoledronic Acid was assessed by detection of unprenylated RAP1A **(K)** Western blot showing the BAP1 protein status (green indicates mutated genotype, red indicates wild-type) of the human uveal melanoma cell lines used for long-term clonogenicity assays. **(L-S)** The quantifications of three independent human uveal melanoma clonogenicity assays as shown in Figure 4B. Data are mean±S.E.M., n = 3 independent experiments.

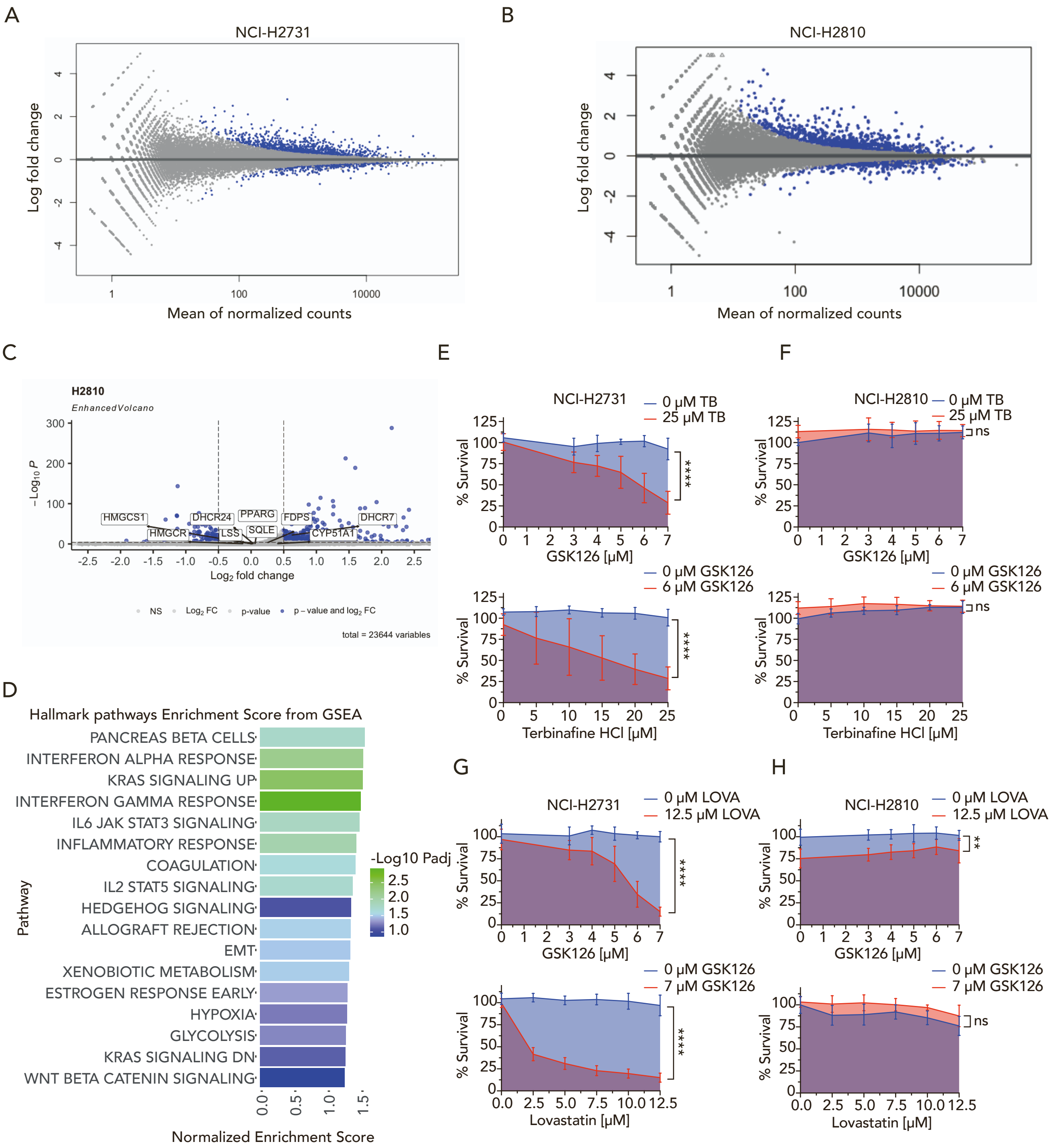
A
NCI-H2731
Log fold change
Mean of normalized counts
B
NCI-H2810
Log fold change
Mean of normalized counts
C
H2810
EnhancedVolcano
HMGCS1
HMGCR
DHCR24
LSS
PPARG
SQLE
FDPS
CYP51A1
DHCR7
Log2 fold change
NS
Log2 FC
p-value
p – value and log2 FC
total = 23644 variables
D
Hallmark pathways Enrichment Score from GSEA
PANCREAS BETA CELLS
INTERFERON ALPHA RESPONSE
KRAS SIGNALING UP
INTERFERON GAMMA RESPONSE
IL6 JAK STAT3 SIGNALING
INFLAMMATORY RESPONSE
COAGULATION
IL2 STAT5 SIGNALING
HEDGEHOG SIGNALING
ALLOGRAFT REJECTION
EMT
XENOBIOTIC METABOLISM
ESTROGEN RESPONSE EARLY
HYPOXIA
GLYCOLYSIS
KRAS SIGNALING DN
WNT BETA CATENIN SIGNALING
Pathway
-Log10 Padj
Normalized Enrichment Score
E
NCI-H2731
0 μM TB
25 μM TB
% Survival
GSK126 [μM]
0 μM GSK126
6 μM GSK126
Terbinafine HCl [μM]
F
NCI-H2810
0 μM TB
25 μM TB
ns
% Survival
GSK126 [μM]
0 μM GSK126
6 μM GSK126
Terbinafine HCl [μM]
G
NCI-H2731
0 μM LOVA
12.5 μM LOVA
% Survival
GSK126 [μM]
0 μM GSK126
7 μM GSK126
Lovastatin [μM]
H
NCI-H2810
0 μM LOVA
12.5 μM LOVA
% Survival
GSK126 [μM]
0 μM GSK126
7 μM GSK126
ns
Lovastatin [μM]

Supplementary Figure S5: RNA sequencing of BAP1-deficient and proficient human mesothelioma cell lines, related to Figure 5

(A) MA-plot showing the distribution of changes in gene expression upon treatment with EZH2 inhibitor versus DMSO control in BAP1-deficient NCI-H2731. **(B)** Likewise, for BAP1-proficient NCI-H2810. **(C)** Volcano plot representing the changes in gene expression of the cholesterol homeostasis genes (labeled) upon EZH2 inhibition treatment in BAP1-proficient NCI-H2810. The x-axis shows log2 fold change (Treated/Control), the y-axis shows the adjusted p values, which were calculated by differential expression test (using the DESeq2 package in R). A gene was considered to be differentially expressed with a p-value < 0.0001 and Log2 fold change > 0.5 (in blue). **(D)** Pathway enrichment within the MSigDB Hallmark pathway database in NCI-H2810, shown are pathways with FDR < 0.25. **(E)** Dose-response curves for BAP1-deficient human mesothelioma cell line NCI-H2731 of increasing concentrations of GSK126 alone or in combination with fixed concentrations of Terbinafine HCl (top), and of increasing concentrations of Terbinafine HCl alone or supplemented with fixed concentrations of GSK126. **(F)** Likewise, for BAP1-proficient human mesothelioma cell lines NCI-H2810. **(G)** Dose-response curves for BAP1-deficient human mesothelioma cell line NCI-H2731 of increasing concentrations of GSK126 alone or in combination with fixed concentrations of Lovastatin (top), and of increasing concentrations of Lovastatin alone or supplemented with fixed concentrations of GSK126. **(H)** Likewise, for BAP1-proficient human mesothelioma cell lines NCI-H2810.

A B

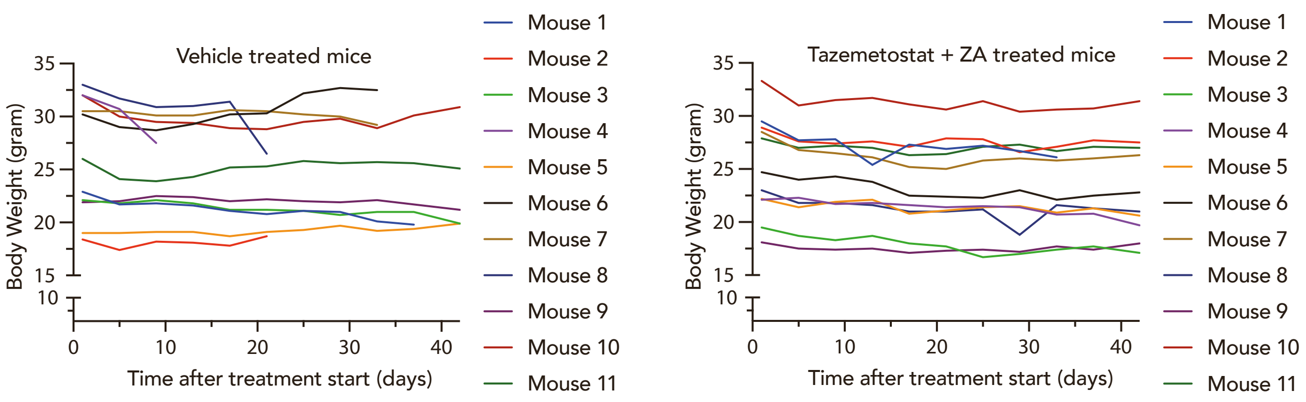

Supplementary Figure S6: Treatment with the combination of Tazemetostat and Zoledronic acid has no effect on animal well-being compared to the control group, related to Figure 6

(A) Graph showing the body weight of all mice in the vehicle-treated group during the duration of the treatment plus two weeks follow up. **(B)** Graph showing the body weight of all mice in the combination-treated group during the duration of the treatment plus two weeks follow up. Graphs indicates no effect of treatment on animal well-being.

Table Methods S1. List of primer sequences used for qPCR and ChIP-qPCR, related to STAR methods

qPCR primers	Forward	Reverse
BAP1	CGATCCATTTGAACAGGAAGA	CTCGTGGAAGATTTCGGTGT
HOXA6	CGCGCAAATGAGTTCCTATT	CATAGCCAGCCTGGTAGAGG
HOXA10	ACACTGGAGCTGGAGAAGGA	TCACTTGTCTGTCCGTGAGG
USP43	GTGGGCATTACACAGCCTACT	GGATGCTGTTCCGCTTCTGA
LSS	GCACTGGACGGGTGATTATGG	TCTCTTCTCTGTATCCGGCTG
PPARG	AGCCTGCGAAAGCCTTTTGGTG	GGCTTCACATTCAGCAAACCTGG
CYP51A1	GAAACGCAGACAGTCTCAAGA	ACGCCCATCCTTGTATGTAGC
FDPS	GTGCTGACTGAGGATGAGATG	GCTCGATCAGGTTCAGGTAATAG
DHCR7	GCAGGGGTTGTGAACAAGTAT	GAGACGGCATAGCCAAGGAT
SQLE	GCGTGCTTGGCTCTGCTTT	CCTGGGCATCAAGACCTTCCA
DHCR24	GCACAGGCATCGAGTCATCAT	GCAGGCTCATCATCAATACGGA
GAPDH	GTCTCCTCTGACTTCAACAGCG	ACCACCCTGTTGCTGTAGCCAA

ChIP qPCR primers	Forward	Reverse
HOXA6	CCCTCTGCAGGACTGTGATTT	CGTTCGGCCATCCAGAAACA
HOXA10	CACTCCCAGTTTGGTTTCGT	CTCCTGGCCCATCAATACAG
USP18	GCGGAACTTCGGTCCCAG	GAACTTGCCTCAAGCGCC
USP43	CCGTAATTTCCACTGCTTGAA	GACTGGGAGTCTTCGTGGTC
